# Admitted Cases of Dengue Fever among Dengue Positive Cases in a Tertiary Care Hospital: A Descriptive Cross-sectional Study

**DOI:** 10.31729/jnma.5307

**Published:** 2021-12-31

**Authors:** Sunita Poudyal, Hem Kumari Subba, Kalpana Sharma

**Affiliations:** 1 School of Nursing, Chitwan Medical College, Bharatpur, Chitwan, Nepal

**Keywords:** *dengue*, *fever*, *profile*

## Abstract

**Introduction:**

Dengue fever, a mosquito-borne disease, is one of the emerging tropical diseases that appear primarily in rainy seasons. The number of dengue cases was increased in recent years in Nepal. Chitwan is one of the risky areas of dengue. This aim of the study is to find out the prevalence of hospital admission among the dengue positive cases.

**Methods:**

The descriptive cross-sectional study was carried out among 323 serologically confirmed dengue fever positive patient admitted in Medicine Inpatients Department of Chitwan Medical College Teaching Hospital. Ethical approval was taken from the Institutional Review Committee (Reference number. 076/077-121 dated August 30, 2019). Data were collected from 1st September 2019 to 31st December 2019 using a structured interview schedule and record review. Convenience sampling was done. Data was analysed using Statistical Package for Social Sciences version 11. Point estimate at 95% Confidence Interval was calculated, with frequency and percentage.

**Results:**

Among 1206 patient with dengue fever, 323 (26.78%) (24.29-29.27 at 95% confidence Interval) were admitted in the tertiary care hospital. Study findings revealed that out of 323 admitted patients with dengue fever, 182 (56.3%) patients were between 20-40 years of age and 179 (55.4%) were males. The highest number of patients were admitted in the months of September 192 (59.4%) and October 101 (31.2%).

**Conclusions:**

Admission rate among dengue positive cases are comparable to other studies of the similar settings. Dengue fever is common among community people especially in young adult and males. Hence, screening of dengue fever in febrile illness is necessary for the early diagnosis and prompt treatment.

## INTRODUCTION

Dengue fever is one of the emerging tropical diseases that appear primarily in rainy seasons. It affects people of all ages and sex, mostly living in endemic areas of warm and moist climates.^1-3^ Severe headache, myalgia, arthralgia, retro-orbital pain, nausea and vomiting are the common symptoms^2,4-6^ requiring hospitalization.^7^ Dengue can be prevented by using personal protective measures against mosquito bites and mosquitoes control measures.^4,5^ In Nepal, dengue was first reported in 2004 from Chitwan^6-8^ and sporadic cases were registered in all the years throughout the country, particularly in Terai region.^9^

Chitwan is the most affected district. The highest numbers of cases were found during the dengue outbreak in 2016 AD.^6^ Studies also showed inadequate awareness regarding dengue among community people of Nepal.^10-12^ Awareness regarding dengue fever, early diagnosis and treatment are essential weapons to fight against dengue outbreaks.

Hence, the aim of the study is to find out the prevalence of hospital admission among the dengue positive cases in a tertiary-care hospital in Chitwan.

## METHODS

A descriptive cross-sectional study was conducted among the patients admitted to the Medicine Department of Chitwan Medical College Teaching Hospital (CMCTH) from 1st September 2019 to 31st December 2019. Ethical approval was obtained from CMC Institutional Review Committee (CMC-IRC) (Reference no. CMC-IRC/076/077-121 dated August 30, 2019) and the data were collected using structured interviews schedule and records reviews. The purpose of the study was explained to the patients, and their informed consent was taken.

Patients with serologically confirmed dengue admitted in different medicine department were selected purposively as the study sample. First, patients who tested positive for dengue either Immunoglobulin M (IgM), or Immunoglobulin G (IgG) or dengue NS1 antigen by ELISA were identified from their records file. Lab parameters were studied from the patients' laboratory reports. Data related to socio-demographic and disease-related information was collected using a structured interview schedule. Patients who were critically ill or unable to communicate or not willing to participate were excluded from the study. Convenience sampling was done.

The sample size was calculated using the formula

n = Z^2^ × p × q / e^2^

  = Z^2^ × p × (1-p) / e^2^

  = (1.96)^2^ × 0.5 × 0.5 / (0.03)

  = 1067

Where,

n= required sample size
Z= 1.96 at 95% Confidence Interval (CI)
p= prevalence of admitted cases of dengue among dengue positive cases is taken as 50%
q= 1-p
e= margin of error 3%

Hence, the minimum calculated sample size was 1067 but we took 1206 samples.

The collected data were coded and entered into IBM SPSS software version 20. Descriptive statistics such as frequency distribution, percentage, mean, standard deviation, median, and were computed to describe socio-demographic characteristics, predisposing factors of dengue, clinical features and lab parameters. Point estimate at 95% Confidence Interval was calculated, with frequency and percentage.

## RESULTS

Out of 4060 patients with febrile illness who were tested for dengue through ELISA during the study period, 1289 (31.75%) were dengue positive. Among 1206 patient with dengue fever, 323 (26.78%) (24.2929.27 at 95% confidence Interval) were admitted in the tertiary care hospital.

Out of 323 patients, more than half, 182 (56.3%) of the patients were from Chitwan district, followed by 54 (16.7%) from Makwanpur, 46 (14.2%) from Nawalparasi and 41 (12.7%) from other districts (Tanahu, Gorkha, Bara, Dhading, Rauthat, Sarlahi). Similarly, the highest numbers 192 (59.4%) of dengue fever patients were admitted during the months of September, followed by 101 (31.2%) in October, 25 (7.7%) in November, and 5 (1.5%) in December.

About 182 (56.3%) patients were between 20 to 40 years. About 179 (55.4%) were male and 144(44.6) were female. 235 (72.8%) were married and 144 (44.6%) had completed secondary level of education (Table 1).

**Table 1:** Socio-demographic Characteristics of the Patients (n= 323).

Variables	n (%)
**Age**	
<20year	51 (15.8)
20-40year	182 (56.3)
40-60year	70 (21.7)
>60year	20 (6.2)
Median age (IQR) - 32(75-25), minimum age- 16 year, maximum- 88years	
**Sex**	
Male	179 (55.4)
Female	144 (44.6)
**Marital status**	
Married	235 (72.8)
Unmarried	88 (27.2)
**Education status**	
Illiterate	28 (8.7)
General read and write	19 (5.9)
Basic level	49 (15.2)
Secondary level	144 (44.6)
Bachelor and above	83 (25.7)

Out of 323 patients, 210 (65.0%) patients had a bushy area around their houses (Table 2). Only 9 (2.8%) patients had a previous history of dengue fever and 42 (13%) had a history of dengue fever in their family. Similarly, only 34 (10.5%) patients had a history of chronic diseases. Among them, 13 (4%) patients were suffering from hypertension and 7 (2.2%) from diabetes mellitus. More than half of the patients 168 (52.0%) had a history of water stagnation inside their houses and surrounding and 210 (65.0%) patients had a bushy area around their houses. Very few patients 49 (15.2%) had a history of travelling to dengue-endemic areas, and 72 (22.3%) had a history of living with DF patients.

**Table 2:** Predisposing Factors of Patients (n= 323).

Predisposing Factors	n (%)
Previous history of dengue	9 (2.8)
History of chronic disease	34 (10.5)
Family history of dengue	42 (13.0)
Water stagnation inside houses and surrounding	168 (52.0)
Bushy area around the houses	210 (65.0)
History of travel to dengue-endemic area	49 (15.2)
History of living with a dengue patient fever	72 (22.3)

Almost all patients had fever 311 (96.6%) followed by headache 263 (81.7%), muscle and joint pain 207 (64.3%), and nausea/vomiting 192 (59.6%) (Table 3).

**Table 3:** Clinical features among patients (n = 323).

Clinical Features	n (%)
Fever	311 (96.6)
Headache	263 (81.7)
Muscle and joint pain	207 (64.3)
Nausea and vomiting	192 (59.6)
General weakness	171 (53.1)
Retro-orbital pain	158 (49.1)
Backache	95 (29.5)
Skin rashes	69 (21.4)
Cough	55 (17.1)
Itching	40 (12.4)
Diarrhea	23 (7.1)
Abdomen distension	20 (6.2)
Abdomen pain	10 (3.1)
Petechiae	8 (2.5)
Others	10 (3.1)

Other features: shortness of breath, Malena, Dizziness.

More than half of patients 171 (52.9%) had platelets count <100000 per microliter of blood, and 208 (64.4%) patients had <4000 cells/μL of total leucocyte count. Similarly, liver enzymes Aspartate Transaminase (AST) and Al-anine Transaminase (ALT) were elevated in 214 (66.3%) and 172 (53.3%) patients respectively (Table 4).

**Table 4:** Lab Parameters among Patients (n=323).

Lab Parameters	n (%)
**Leucocyte count**	
<4000 (cells/μL)	208 (64.4)
≥4000 (cells/μL)	115 (35.6)
**Platelets**	
<100000 per microliter of blood	171 (52.9)
≥100000 per microliter of blood	152 (47.1)
**AST**	
>37 IU/L	214 (66.3)
≤37 IU/L	109 (33.7)
**ALT**	
>42 IU/L	172 (53.3)
≤42 IU/L	151 (46.7)

Out of 323 patients, only 6 (1.9%) patients had suffered from complications, and the common complication was pleural effusion 3(0.9%). Moreover, 187 (57.9%) patients stayed in the hospital for less than three days, and the mean duration of hospital stay was 3.52±1.57 days. Almost all patients 320 (99.1%) were cured and discharged from the hospital, and only 0.3% death was recorded.

## DISCUSSION

In this study, the most affected age group was 20-40 years 56.3%, and more than half 55.4% were male. A similar finding was reported by Gupta, et al.^13^ where adults between 25 and 40 years of age were mostly infected by the dengue virus. Likewise, other studies in India^2,14^ and Pakistan^15^ reported similar results. However, Savargaonkar, et al.^16^ found 11-30 years as the most commonly afflicted age group. Most of these studies indicate young adults as the most affected groups. These might be due to the fact that these age groups are the active working population and are most likely to be exposed to mosquitoes because of their frequent outdoor activities.

The prevalence of dengue was recognized in different geographical areas with different climatic conditions. In this study, the highest percentages of dengue fever patients were seen in the months of September 59.4%, followed by October 31.2% and November 7.7%. This is almost consistent with the findings of the studies in which dengue cases were seen from the month of June to September, with a maximum rate of positivity in August^17^ and September.^6^ Likewise, other studies showed that the cases of dengue were seen from August^2,15^ with a peak in October and November.^15^

Almost all patients 96.6% were presented with a history of fever. Consistent findings were reported by the studies of India and Nepal, where most common symptom was fever 100%.^5,6^ Moreover, common clinical symptoms of dengue in our study were headache 81.7%, muscle and joint pain 64.3%, nausea vomiting 59.6%, general weakness 53.1% and retro-orbital pain 49.1%, whereas rashes 21.4% and abdominal pain (3.1) were observed less frequently. These are consistent with the findings of other studies which reported headache, myalgia, vomiting, arthralgia, abdominal pain, rashes, retro-orbital pain, vomiting, and joint pain as the symptoms of dengue fever.^2,5,6,14^

In our study, more than half of patients (52.9%) had thrombocytopenia (platelets <100000 per microliter of blood) and 64.4% had leucopenia (<4000cells/μL of total leucocyte count). This finding is supported by other studies which reported that thrombocytopenia is the most common abnormal laboratory finding followed by leucopenia.^5,6,18^ However, Chitkara, et al.^2^ found higher percentages (93.1%) of dengue fever patients with thrombocytopenia and lower percentage (43%) with leucopenia.

Regarding the length of hospital stay, more than half (57.9%) of this study's patients were stayed in the hospital for less than three days and the mean hospital stay was 3.52±1.57days. This finding suggests that patients' condition improves quickly, allowing them to be discharged sooner. A similar result was reported by the study of Thaher, et al.^14^ Survival after dengue fever is high among patients, and most of them (99.1%) were cured and discharged from the hospital, and the death rate was only 0.3%. A study of Pakistan^15^reported a 0.5% death rate of cases in the tertiary care hospitals, whereas a 4% mortality rate reported in Gujarat, India.^5^

## CONCLUSIONS

Admission rate among dengue positive cases are comparable to other studies of the similar settings. Dengue fever is common among young adults, and males are the most susceptible group for dengue fever. Fever, headache and myalgia are the most common presenting symptoms. Therefore, screening of dengue fever in patients with febrile illness is necessary for the early diagnosis and prompt treatment. Furthermore, awareness campaigns are required to protect people against dengue and to limit future spread of dengue in other areas.
